# Supplementary material for: Coexistence of SOS-Dependent and SOS-Independent Regulation of DNA Repair Genes in Radiation-Resistant Deinococcus Bacteria
Source: Cells. 2021 Apr 16;10(4):924. doi: 10.3390/cells10040924 (PMC8072749; doi:10.3390/cells10040924)
Supplement: Supplementary file 1 [file cells-10-00924-s001.zip › cells-1186292.pdf]

Supplementary Table for:
Coexistence of SOS-dependent and SOS-independent regulation of DNA repair genes in radiation-resistant *Deinococcus* bacteria
Laurence Blanchard and Arjan de Groot

Supplementary Table S1. Accession numbers of the proteins encoded by *lexA-imuY-imuB-C-dnaE2* cassettes in *Deinococcus* bacteria.

species	LexA	ImuY	ImuB-C	DnaE2
D. deserti	WP_012694770.1	WP_012694771.1	WP_012694772.1	ACO47650.1
D. aerolatus	WP_188974186.1	WP_188974185.1	WP_188974184.1	WP_188974183.1
D. aquiradiocola	WP_188961532.1	WP_188961531.1	WP_188961530.1	WP_188961711.1
D. budaensis	WP_184027903.1	WP_184027906.1	WP_184027909.1	WP_184027912.1
D. cellulosilyticus	WP_146887394.1	WP_146887392.1	WP_146887390.1	WP_186816141.1
D. cellulosilyticus	WP_146891023.1	WP_146891026.1	WP_146891029.1	WP_146891032.1
D. daejeonensis	WP_189059405.1	WP_189059403.1	WP_189059400.1	WP_189059460.1
D. daejeonensis	WP_119672580.1	WP_189056591.1	WP_119672233.1	WP_189056413.1
D. ficus 2009	WP_081425875.1	WP_022800705.1	WP_022800706.1	WP_022800707.1
D. ficus 2009	WP_022803141.1	WP_022803140.1	WP_022803139.1	WP_028306285.1
D. ficus CC-FR2-10	WP_081425875.1	WP_027463211.1	WP_043778331.1	WP_043778329.1
D. ficus CC-FR2-10	WP_027464396.1	WP_027464397.1	WP_027464398.1	WP_027464399.1
D. ficus CGMCC 1.18438	WP_191241408.1	WP_191241360.1	WP_191241359.1	WP_191241358.1
D. grandis	WP_058979539.1	WP_058979537.1	WP_058979535.1	WP_083524274.1
D. grandis	WP_058979356.1	WP_058979066.1	WP_058979068.1	WP_058979070.1
D. humi	WP_184135053.1	WP_184135056.1	WP_184135059.1	WP_184135062.1
D. humi	WP_184137688.1	WP_184137686.1	WP_184137684.1	WP_184137682.1
D. indicus	WP_055362527.1	WP_055362526.1	WP_055362525.1	WP_055362522.1
D. indicus	WP_191301194.1	WP_191301185.1	WP_191301183.1	WP_191301181.1
D. koreensis	WP_103311688.1	WP_103311689.1	WP_103311690.1	WP_103311691.1
D. koreensis	WP_103314225.1	WP_103314224.1	WP_103314223.1	WP_103314222.1
D. malanensis	WP_189010110.1	WP_189010108.1	WP_189010106.1	WP_189010105.1
D. marmoris DSM 12784	WP_029483416.1	WP_029483417.1	WP_029483418.1	WP_051655457.1
D. marmoris PAMC 26562	WP_075834676.1	WP_075834674.1	WP_075834672.1	WP_139323019.1
D. metalli	WP_184114207.1	WP_184114304.1	WP_184114205.1	WP_184114203.1
D. metalli	WP_191240085.1	WP_184114723.1	WP_184114724.1	WP_184114726.1
D. peraridilitoris	WP_015236734.1	WP_015236735.1	WP_015236736.1	WP_015236737.1
D. radiopugnans	WP_139404716.1	WP_139404715.1	WP_139404714.1	WP_139404713.1
D. radiotolerans	WP_189070213.1	WP_189070212.1	WP_189070211.1	WP_189070209.1
D. roseus	WP_189009241.1	WP_189009226.1	WP_189009229.1	WP_189009232.1
D. roseus	WP_189005274.1	WP_189005275.1	WP_189005276.1	WP_189005277.1
D. ruber	WP_189091652.1	WP_189091653.1	WP_189091654.1	WP_189091656.1
D. saudiensis	WP_188845450.1	WP_188845448.1	WP_188845446.1	WP_188845442.1
D. saudiensis	WP_188846919.1	WP_188846920.1	WP_188846921.1	WP_188846922.1
D. sedimenti	WP_189074292.1	WP_189074291.1	WP_189074290.1	WP_189074289.1
D. xianganensis	MXV18662.1	MXV18663.1	MXV18664.1	MXV18665.1
D. yavapaiensis	PYE56319.1	PYE56320.1	PYE56321.1	PYE56322.1

The strain number is mentioned only when more than one strain of a species is sequenced. The cassettes in the different bacteria were found using protein BLAST (blastp) at https://blast.ncbi.nlm.nih.gov/Blast.cgi with default settings and with *D. deserti* ImuY as query (BLAST searches performed February 2021); then the upstream and downstream regions of the identified *imuY* homologs were analyzed, which showed that these *imuY* genes are directly adjacent to *lexA*, *imuB-C* and *dnaE2* as in *D. deserti*.
